# The role of pharmacists in quality management of venous thromboembolism: a retrospective, observational, single-center study in the cardiothoracic surgery department

**DOI:** 10.3389/fphar.2026.1819628

**Published:** 2026-06-26

**Authors:** Sha Qiu, Daiyi Li, Bangqin Hu, Qiao Ling, Yan Qian

**Affiliations:** Department of Pharmacy, The Second Affiliated Hospital of Chongqing Medical University, Chongqing, China

**Keywords:** clinical pharmacist, management, prophylaxis, quality evaluation, venous thromboembolism

## Abstract

**Background:**

Venous thromboembolism (VTE) prophylaxis frequently falls short of ideal standards in current clinical practice. Based on China’s policy-oriented quality evaluation indicators for VTE management, this study explored the role of pharmacists in VTE prophylaxis quality management and conducted preliminary verification among cardiothoracic surgery inpatients.

**Methods:**

We retrospectively analyzed patients hospitalized in the cardiothoracic surgery department of our hospital between 1 January 2025 and 30 June 2025. The primary outcome measures included the implementation rates of pharmacological, mechanical, combined, and standardized prophylaxis. The secondary outcomes included the incidence of hospital-associated VTE, in-hospital major bleeding events, and the mortality attributable to hospital-associated VTE. Patients were categorized into the baseline group, pilot intervention group, and pharmacist intervention group based on the time series and the extent of pharmacist involvement. We compared the outcome measures for each group within both the original study cohort and the propensity score matching (PSM) cohort. We also performed stratified analyses by baseline VTE risk category (moderate vs. high risk) to assess potential effect modification.

**Results:**

A total of 1813 patients were enrolled in this study, with a mean age of 62.1 ± 11.8 years. Among them, 1171 (64.6%) were identified as being at moderate or high risk of VTE. Progressive pharmacist intervention coincided with increases in the implementation of mechanical prophylaxis (54.6% vs. 74.2% vs. 79.7%, p < 0.001, Cramer’s V = 0.224, 95% CI [0.182, 0.266])) and standardized prophylaxis (57.1% vs. 74.8% vs. 77.8%, p < 0.001, Cramer’s V = 0.188, 95% CI [0.146, 0.228]) compared to the baseline period. Conversely, no statistically significant alterations were observed in the rates of pharmacological (43.9% vs. 45.7% vs. 49.1%, p = 0.351) and combined prophylaxis implementation (70.7% vs. 70.3% vs. 69.5%, p = 0.976). This finding was corroborated by the PSM cohort. The reduction in hospital-associated VTE were observed, though the analysis was based on very few events (9 vs. 1), which limits interpretability.

**Conclusion:**

The pharmacist-participated VTE management model was associated with improved implementation rates of mechanical and standardized prophylaxis, generating hypotheses about its potential utility in optimizing quality control for in-hospital VTE prevention and management, which remain to be validated through concurrent controlled trials.

## Introduction

Venous thromboembolism (VTE) encompassing deep vein thrombosis (DVT) and pulmonary embolism (PE), has emerged as the third most prevalent cardiovascular and cerebrovascular disease, following ischemic heart disease and stroke (Gregson et al., 2019). An analysis of data from 2007 to 2016 revealed that the hospitalization rate for VTE in China rose from 3.2 per 100,000 people to 17.5 per 100,000 people over the past decade, becoming a serious issue for clinical medical staff and hospital administrators (Martin et al., 2025). As a significant risk factor for hospital-related deaths and disabilities, VTE possesses a preventable characteristic (Roberts et al., 2017). Timely and effective prophylaxes could significantly reduce VTE events and associated complications (Lutsey and Zakai, 2023). However, a survey indicated that the adoption rate of appropriate VTE prophylaxis in China is only 9.3% in surgical departments and 6.0% in internal medicine departments (Zhai et al., 2019), significantly lower than the international VTE prophylaxis rate ranging from 39.5% to 58.5% (Cohen et al., 2008). In recent years, China has actively explored ways to further standardize the management of VTE (Cohen et al., 2008; Zhang et al., 2022). The National Health Commission of China has explicitly identified improving the standardized prophylaxis rate of VTE as one of the ten major goals for improving national medical quality and safety. Subsequently, the specific indicators for evaluating the quality of VTE management have been further refined and clarified in 2022 (Expert Committee of the National Capacity Building Project for the Prevention and Treatment of Pulmonary Embolism and Deep Vein Thrombosis, 2022), and have since been gradually incorporated into the performance management systems of domestic hospitals. Although some studies have shown that pharmacist involvement in VTE management might effectively reduce the incidence of VTE in patients (Bauer et al., 2008; Cronin et al., 2009; Expert Committee of the National Capacity Building Project for the Prevention and Treatment of Pulmonary Embolism and Deep Vein Thrombosis, 2022; Wang et al., 2025), relevant research remains limited in China. As an exploratory clinical observation, this study analyzed the actual role and clinical influence of pharmacist participation. Using cardiothoracic surgery inpatients as subjects, we conducted relevant practice observation for the first time based on national standardized quality indicators of VTE management.

## Methods

### Study population

From 1 January 2025, to 30 June 2025, a retrospective, sequential observational, single-center study was conducted among inpatients admitted to the cardiothoracic surgery department (Ethics Registration Number: NO. 2025–55). The study included inpatients who (1) had been admitted to the cardiothoracic surgery department for ≥3 days; (2) had complete electronic medical records. Patients were excluded if they (1) had been diagnosed with VTE upon admission; (2) required long-term antithrombotic therapy due to conditions such as cerebral infarction, atrial fibrillation, coronary heart disease, or valve replacement surgery. Based on the time series and the intensity of pharmacist involvement, patients were categorized into three groups: baseline group (January-February 2025, without pharmacist involvement), pilot intervention group (March-April 2025, with initial pharmacist intervention), and pharmacist intervention group (May-June 2025, with comprehensive pharmacist management).

### Outcome measures

The primary outcomes were VTE prophylaxis quality indicators, encompassing the implementation rates of pharmacological, mechanical, combined, and standardized prophylaxis. The secondary outcomes, as quality indicators of the outcome, included the hospital-associated VTE incidence, the major bleeding events incidence during hospitalization, and the mortality of hospital-associated VTE. The standardized prophylaxis was defined as the proportion of discharged patients with a VTE risk assessment indicating moderate or high risk who actually received standardized prophylaxis. Both this measure and the diagnosis of VTE were assessed based on the “Chinese Guidelines for the Prevention and Treatment of Thrombotic Diseases” (Expert Committee of the “Chinese Guidelines for the Prevention and Treatment of Thrombotic Diseases, 2018). Major bleeding events were defined by the criteria of the International Society on Thrombosis and Haemostasis (Schulman et al., 2010). The calculation methods for various indicators were based on the “Quality Evaluation and Management Guidelines for the Prevention and Treatment of Venous Thromboembolism in Hospitals (2022 Edition)” (Expert Committee of the National Capacity Building Project for the Prevention and Treatment of Pulmonary Embolism and Deep Vein Thrombosis, 2022), as specified in Supplementary Appendix Table 1. All groups of patients were followed up until they were discharged, transferred to other departments, or deceased.

### The role of pharmacists in the quality management of VTE prophylaxis within our hospital

Since 1 March 2025, six professionally trained anticoagulation-specialized clinical pharmacists participated in the hospital-wide VTE management pilot program initiated by the Medical Affairs Department. In this pilot phase, targeted interventions were implemented in poorly performing departments, with intervention intensity yet to be standardized and quantified.

From 1 May 2025 onwards, the formal pharmacist-participated VTE management model was fully implemented with oversight from the Medical Affairs Department. Intervention intensity was uniformly standardized and quantitatively specified, with clear job responsibilities formulated. The standardized intervention protocols were detailed below: (1) Regular training: Monthly centralized training on standardized VTE prevention was conducted for clinical medical and nursing staff. (2) On-site daily review and real-time intervention: The VTE Intelligent Decision Support System (VTE-IDSS) (V3.0–20220932, Dr. Breath. Com, Beijing, China) automatically completed VTE risk assessment via the Caprini (2005) scale and Padua scale (Barbar et al., 2010), together with bleeding risk assessment (Expert Committee of the Thrombosis and Vascular Special Fund, 2018) at key clinical time nodes. Clinical pharmacists conducted systematic daily case reviews of all hospitalized patients in designated departments, focusing on verifying the rationality of prophylaxis and pharmacological regimens for patients at moderate or high VTE risk. Timely face-to-face communication with attending physicians was completed within the same working day for unreasonable regimens. Meanwhile, standardized medication education and regular pharmaceutical monitoring were provided for patients receiving anticoagulant prophylaxis. (3) Monthly summary and quality improvement: Pharmacists conducted quantitative statistical analysis on all VTE management quality indicators on a monthly basis, and assisted clinical departments in formulating targeted rectification and optimization measures steadily.

In brief, intervention intensity was gradually escalated from unquantified exploratory practice in the pilot stage to standardized, quantifiable and full-coverage professional intervention in the formal stage.

### Statistical analyses

All statistical analyses were conducted in Python 3.12.4. Continuous variables were presented as mean ± standard deviation and were compared using the t-test (after passing the homogeneity of variance test) or non-parametric test (if variance was not homogeneous). Categorical variables were expressed as numbers and percentages and were compared using the chi-square test or Fisher’s exact test, as appropriate. Every pair of groups within the three groups were compared. To minimize biases and confounding variables among samples, Propensity score matching (PSM) was conducted separately for two pairwise comparisons at a 1:1 ratio without replacement, using a caliper width of 0.05. Matching variables included sex, age, comorbidities, VTE risk stratification, bleeding risk stratification, and surgical status during hospitalization. The balance after matching was assessed using the standardized mean difference (SMD), with an absolute value < 0.2 meeting the balance criteria. Comparative analyses were conducted in both the original full cohort and the matched cohort. Moreover, stratified analyses by baseline VTE risk category were performed (moderate vs. high risk) to assess potential effect modification. To account for multiple comparisons, P-values were adjusted using the Bonferroni correction, and statistical significance was set at P < 0.05.

## Results

### Patient characteristics

**FIGURE 1:**
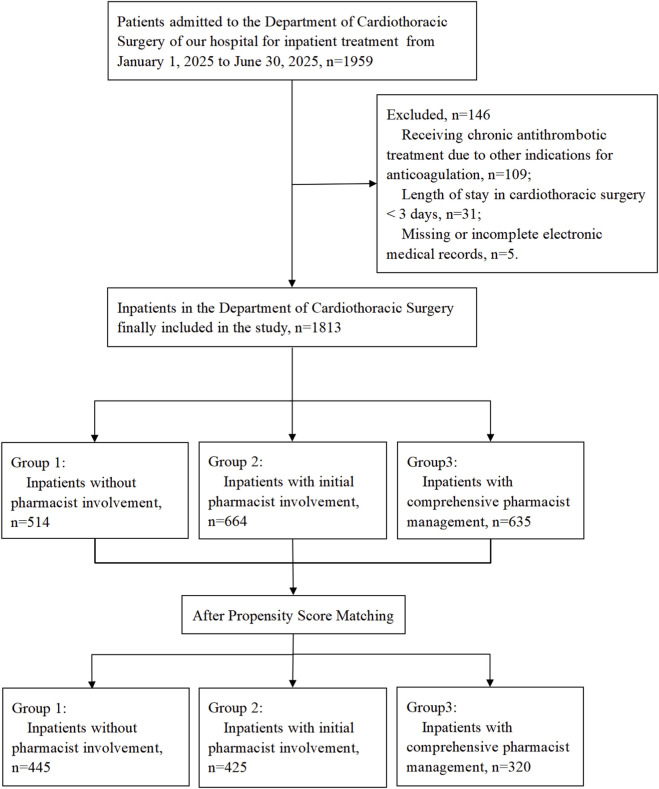
The flow diagram of selection of eligible patients.

From 1 January 2025, to 30 June 2025, a total of 1959 patients were admitted to the cardiothoracic surgery department of our hospital for inpatient treatment. However, 146 patients were excluded due to the reasons outlined in Figure 1. Ultimately, 1813 patients met the inclusion criteria, with 514 in the baseline group, 664 in the pilot intervention group, and 635 in the pharmacist intervention group. A summary of the baseline characteristics of the enrolled patients is provided in Table 1. The average age of the patients was 62.1 ± 11.8 years, with 696 female patients (38.4%) and 1171 patients at moderate or high risk of VTE (64.6%). The baseline characteristics of the three groups were relatively comparable, except for age and certain cardiovascular comorbidities such as hypertension and heart failure. After PSM, the baseline characteristics were well balanced, with 445 cases in the baseline group, 425 cases in the pilot intervention group, and 320 cases in the pharmacist intervention group. The balance test before and after matching is presented in Supplementary Appendix Table 2.

**TABLE 1:** Demographics and characteristics of patients before and after propensity score matching.

Variables	Original groups (n = 1813)					Matched groups (n = 1190)				
	Baseline group (n = 514)	Pilot intervention group (n = 664)	Pharmacist intervention group (n = 635)	F/Chi-square value	*P* value	Baseline group (n = 445)	Pilot intervention group (n = 425)	Pharmacist intervention group (n = 320)	F/Chi-square value	*P* value
Age, years	61.0 ± 12.3	62.7 ± 10.9	62.4 ± 12.1	3.2	0.042	60.9 ± 12.5	62.4 ± 11.0	61.2 ± 12.0	1.8	0.164
Sex, n (%)										
Female	192 (37.4)	270 (40.7)	234 (36.9)	2.3	0.314	180 (40.4)	177 (41.6)	131 (40.9)	0.1	0.937
Male	322 (62.6)	394 (59.3)	401 (63.1)	​	​	265 (59.6)	248 (58.4)	189 (59.1)	​	​
Risk assessment levels of VTE, n (%)										
Low risk	183 (35.6)	251 (37.8)	208 (32.8)	9.3	0.053	162 (36.4)	157 (36.9)	107 (33.4)	4.3	0.364
Moderate risk	214 (41.6)	284 (42.8)	260 (40.9)	​	​	177 (39.8)	176 (41.4)	123 (38.4)	​	​
High risk	117 (22.8)	129 (19.4)	167 (26.3)	​	​	106 (23.8)	92 (21.7)	90 (28.1)	​	​
Risk assessment level of bleeding, n (%)										
Low risk	511 (99.4)	660 (99.4)	632 (99.5)	0.1	0.945	442 (99.3)	421 (99.1)	318 (99.4)	0.3	0.858
High risk	3 (0.6)	4 (0.6)	3 (0.5)	​	​	3 (0.7)	4 (0.9)	2 (0.6)	​	​
Prepare to perform the surgery during hospitalization, n (%)										
No	292 (43.2)	323 (51.4)	322 (49.3)	8.1	0.017	225 (50.6)	190 (44.7)	150 (46.9)	3.1	0.217
Yes	222 (56.8)	341 (48.6)	313 (50.7)	​	​	220 (49.4)	235 (55.3)	170 (53.1)	​	​
Combined cardiovascular diseases, n (%)										
Arrhythmia	83 (16.1)	109 (16.4)	130 (20.5)	4.9	0.085	74 (16.6)	83 (19.5)	62 (19.4)	1.5	0.474
Hypertension	117 (22.8)	123 (18.5)	166 (26.1)	10.9	0.004	104 (23.4)	92 (21.6)	75 (23.4)	0.5	0.788
Heart failure	100 (19.5)	100 (15.1)	141 (22.2)	11.0	0.004	88 (19.8)	77 (18.1)	66 (20.6)	0.8	0.672

VTE: venous thromboembolism.

### The involvement of pharmacists in the review and intervention of VTE prophylaxis

On 1 March 2025, pharmacists initially intervened in the management of VTE and explored the mode. From 1 March 2025, to 30 June 2025, pharmacists reviewed preventive measures for 1493 person-times and intervened 399 times for inpatients with moderate-to-high VTE risk in the cardiothoracic surgery department, as shown in Supplementary Appendix Table 3. As evident from the table, the intensity of review and intervention by pharmacists gradually increased with their deeper participation.

### The quality of VTE prophylaxis

**FIGURE 2:**
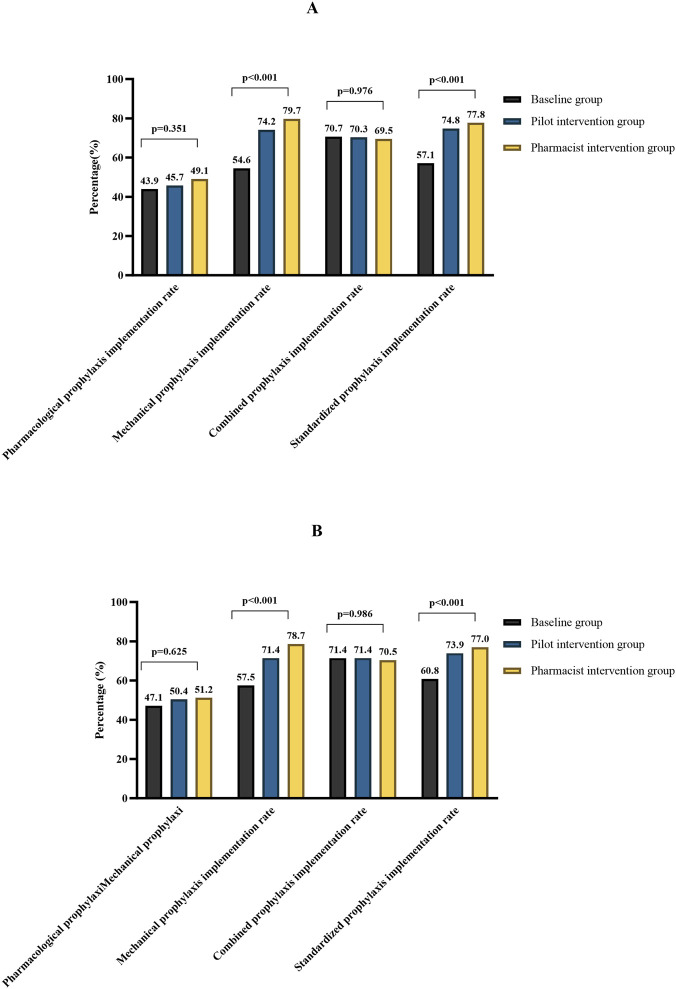
The quality of VTE prophylaxis in original groups **(A)** and matched groups **(B)**. Black column represents the percentage in baseline group, blue column represents the percentage in pilot intervention group and yellow column represents the percentage in pharmacist intervention group.

The quality of VTE prophylaxis is presented in Figure 2. Implementation rates of mechanical prophylaxis (54.6% vs. 74.2% vs. 79.7%, p < 0.001, Cramer’s V = 0.224, 95% CI [0.182, 0.266]) and standardized prophylaxis (57.1% vs. 74.8% vs. 77.8%, p < 0.001, Cramer’s V = 0.188, 95% CI [0.146, 0.228]) varied significantly across the three study periods. A gradual upward trend was noted over time (Figure 2A). The results from the PSM cohort were in line with the primacy results (Figure 2B). Notably, mechanical prophylaxis was a core component of the standardized VTE prophylaxis bundle for moderate-risk patients, so changes in these two measures are closely correlated. Concurrent non-intervention departments showed no parallel improvement in VTE process metrics (Supplementary Appendix Table 4). Although the implementation rate of pharmacological prophylaxis gradually increased, there was no significant statistical difference (43.9% vs. 45.7% vs. 49.1%, p = 0.351). The implementation rate of combined prophylaxis showed no significant change (70.7% vs. 70.3% vs. 69.5%, p = 0.976). Stratified analysis revealed significant gains in both mechanical and standardized prophylaxis among moderate-risk patients; conversely, after Bonferroni correction, no significant differences were observed in high-risk patients (Table 2).

**TABLE 2:** Stratified analysis of VTE prophylaxis quality by VTE risk category (Moderate vs. High).

Indicators	Original groups (n = 1813)							Matched groups (n = 1190)						
	Baseline group (n = 514)	Pilot intervention group (n = 664)	Pharmacist intervention group (n = 635)	Chi-square value	P value	Cramér’s V, 95% CI	Corrected *P*-value	Baseline group (n = 445)	Pilot intervention group (n = 425)	Pharmacist intervention group (n = 320)	Chi-square value	P value	Cramér’s V, 95% CI	Corrected *P*-value
Pharmacological prophylaxis implementation rate (%)														
Moderate risk of VTE	25.9	31.8	32.3	0.8	0.155	​	​	28.6	35.4	30.9	1.0	0.207	​	​
High risk of VTE	76.7	76.6	75.6	1.0	0.888	​	​	78.1	79.1	79.6	0.9	0.861	​	​
Mechanical prophylaxis implementation rate (%)														
Moderate risk of VTE	40.1	67.8	73.1	0.4	<0.001	0.279, 95% CI [0.236, 0.318]	<0.001	42.9	64.0	69.9	0.5	<0.001	0.226, 95% CI [0.171, 0.280]	<0.001
High risk of VTE	81.0	88.3	90.2	0.6	0.033	0.091, 95% CI [0.045, 0.137]	0.100	81.9	85.7	90.9	0.6	0.095	​	​
Combined prophylaxis implementation rate (%)														
Moderate risk of VTE	20.3	25.8	26.5	0.8	0.128	​	​	21.7	28.6	26.0	0.9	​	​	​
High risk of VTE	70.7	70.3	69.5	1.0	0.895	​	​	71.4	71.4	70.5	1.0	>0.999	​	​
Standardized prophylaxis implementation rate (%)														
Moderate risk of VTE	46.3	73.9	78.9	0.4	<0.001	0.288, 95% CI [0.244, 0.330]	<0.001	50.3	71.0	74.8	0.5	<0.001	0.218, 95% CI [0.163, 0.272]	<0.001
High risk of VTE	76.9	76.7	76.1	1.0	0.888	​	​	78.3	79.4	80.0	0.9	0.861	​	​

VTE: venous thromboembolism; CI: confidence interval.

### The quality of VTE prophylaxis outcomes

The quality of VTE prophylaxis outcomes is presented in Table 3. In the baseline group and the pharmacist intervention group, 9 patients (1.8%) and 1 patient (0.2%), respectively (p = 0.002, Cramer’s V = 0.097, 95%CI [0.051, 0.143]), were diagnosed with VTE during hospitalization. No major bleeding events or VTE-related deaths were observed in any group. Given the sparse events and limited statistical power, these findings were interpreted cautiously as exploratory data.

**TABLE 3:** Comparison of outcome quality indicators among baseline group, pilot intervention group and pharmacist intervention group.

Outcomes	Baseline group (n = 514)	Pilot intervention group (n = 664)	Pharmacist intervention group (n = 635)	*P* value	Cramér’s V, 95% CI	Corrected *P*-value
Hospital-associated VTE, n (%)	9 (1.8)	0	1 (0.2)	0.002	0.097, 95% CI [0.051, 0.143]	0.005
Major bleeding events, n (%)	0	0	0	NA	NA	NA
Hospital-associated VTE mortality, n (%)	0	0	0	NA	NA	NA

NA: not applicable.

## Discussion

### Major findings

This study aimed to explore the specific role that pharmacists could play in VTE management, guided by national VTE prophylaxis quality indicators under domestic policies. Preliminary validation was conducted in 1813 inpatients from the cardiothoracic surgery department as a pilot example, offering novel insights into VTE management. In this study, pharmacists were involved in various aspects of VTE management, including pre-training, in-process review and intervention, and post-analysis. We found that among hospitalized patients at moderate or high VTE risk, gradual increases in the implementation rates of mechanical and standardized VTE prophylaxis were observed during the period of pharmacist-led whole-process management. This effect remained consistent in both the original cohort and the PSM cohort. Although statistically significant, the hospital-associated VTE reduction (9 vs. 1 events) was underpowered and statistically fragile, precluding robust outcome-level conclusions.

### The quality indicators of VTE management

Due to the characteristics of VTE, such as high incidence, significant harmfulness, and a high rate of missed diagnoses, the prevention and treatment of VTE have been increasingly emphasized globally (Liederman et al., 2020). Although multiple VTE prevention and treatment guidelines have been issued both domestically and internationally, the overall level and quality of VTE prevention and treatment in clinical practice still require improvement. There were ample evidences indicating that using performance indicators as a public accountability mechanism could effectively accelerate the clinical translation and application of evidence-based guidelines (Groce, 2007; Michota, 2007; Amin and Deitelzweig, 2009). Currently, multiple institutions, including the Surgical Care Improvement Project (SCIP), the National Quality Forum (NQF), and the Joint Commission, have established VTE quality assessment indicators to promote continuous improvement in VTE management (Amin and Deitelzweig, 2009; Bratzler, 2010; Dobesh et al., 2013). These indicators are primarily categorized into two types: process measures and outcome measures. Process measures include VTE prophylaxis rate, proportion of patients receiving standardized VTE prophylaxis from 24 h before to 24 h after surgery, proportion of VTE patients receiving anticoagulant bridging therapy, and VTE discharge guidance. Outcome measures encompass the incidence of potentially preventable 5TE. Previously, China lacked a standardized quality control and management system for VTE management. It was not until the relevant guidelines were issued in 2022 that specific indicators for evaluating the quality of VTE management were further clarified (Expert Committee of the National Capacity Building Project for the Prevention and Treatment of Pulmonary Embolism and Deep Vein Thrombosis, 2022). A three-core indicator system focusing on assessment quality, prophylaxis quality, and outcome quality was established for VTE management. The assessment quality indicators encompass VTE risk assessment rate, proportion of patients with moderate-to-high VTE risk, bleeding risk assessment rate, and proportion of high bleeding risk patients. Prophylaxis and outcome quality indicators were detailed in the Outcome measures section, with the latter additionally including standardized treatment rate for hospital-associated VTE. In recent years, Chinese medical institutions have gradually incorporated these quality evaluation indicators into their performance management systems (Zhen et al., 2022).

### The role of pharmacists in the quality management of VTE within hospitals

Limited evidence regarding pharmacist engagement in VTE prevention has accumulated across diverse clinical settings, yet heterogeneity in outcome metrics and intervention intensities precludes direct synthesis. In orthopedic surgery, pharmacist-led management reduced VTE incidence from 7.8% to 2.7% and increased pharmacological prophylaxis among high-risk patients from 33.9% to 51.6% (Wang et al., 2025), demonstrating measurable effects on hard clinical endpoints. Similarly, in a nephrology ward, pharmacist’s intervention increased prophylaxis coverage from 34.8% to 100% among high-VTE-risk patients with renal impairment (Nikvarz and Seyedi, 2022). Collectively, these studies established that pharmacist involvement could improve drug prophylaxis and clinical outcomes.

Conversely, this study observed a divergent trend. Prior relevant investigations predominantly focused on pharmacological prophylaxis while neglecting institution-level process quality indicators, including mechanical, standardized, and combined prophylaxis implementation rates. The present study filled this research gap by incorporating these policy-aligned systematic metrics, and continuous improvements were observed in compliance with mechanical prophylaxis (from 54.6% to 79.7%) and standardized VTE prophylaxis (from 57.1% to 77.8%) alongside the delivery of pharmacist intervention. However, pharmacological and combined prophylaxis rates remained static, diverging from the positive drug-prescribing effects consistently reported in prior literature. Several interrelated mechanisms could explain this discrepancy. First, risk-stratification-driven guideline adherence: the cardiothoracic surgical cohort comprised a notably high proportion of patients at moderate VTE risk, for whom mechanical prophylaxis was the preferred strategy according to the guidelines adopted by our hospital. Stratified analysis confirmed that the marked increase in mechanical prophylaxis was driven primarily by this moderate-risk subgroup (from 40.1% to 73.1%), with only a modest concurrent rise in high-risk patients (from 81.0% to 90.2%). Among moderate-risk patients, the increase in standardized prophylaxis implementation, driven solely by mechanical prophylaxis uptake, coupled with a static pharmacological prophylaxis rate, indicated appropriate guideline-concordant practice rather than implementation failure. Second, clinicians’ risk aversion: among high-risk patients, where pharmacological prophylaxis is formally indicated, the absence of improvement signals persistent implementation barriers. Clinicians frequently prioritize avoiding life-threatening bleeding over thrombotic risk reduction, concerned more with bleeding severity than baseline bleeding probability, particularly in China (Liang et al., 2019; Zhai et al., 2019; Jiang et al., 2024; Wong, 2016). Third, implementation lag: entrenched prescribing habits and the relatively short observation duration likely impeded the full manifestation of pharmacist effects on pharmacological prophylaxis standardization, consistent with the implementation-effectiveness gap described in the quality improvement literature on process-oriented evaluations (Zhang et al., 2025; Lee et al., 2017; Pelletier, 2021). Collectively, these findings suggested that the intervention successfully shifted practice toward guideline-concordant, risk-appropriate prophylaxis for the predominant moderate-risk stratum, but encountered a hard ceiling in expanding pharmacological prophylaxis beyond its pre-existing, risk-selective baseline.

### Strengths and limitations

This study had four principal strengths. First, it employed propensity score matching to balance baseline characteristics in evaluating pharmacist-led interventions within a pragmatic, non-randomized setting. Second, it provided an empirically grounded demonstration of the implementation-effectiveness gap in VTE prevention, generating testable hypotheses regarding the necessary conditions for translating process improvements into pharmacological prophylaxis gains. Third, it was the first to use nationally standardized, policy-oriented quality indicators as primary evaluative endpoints for pharmacist-led VTE management, ensuring direct relevance to healthcare policy and administrative benchmarking. Fourth, it offered feasibility evidence and effect-boundary delineation for consultative, audit-based pharmacist models in settings where clinical pharmacy integration is nascent. Fourth, it provided feasibility evidence and delineated the effect boundaries of Pharmacist-participated VTE quality management model in settings where clinical pharmacy services remain underdeveloped.

This study also had several limitations. First, its retrospective observational design may have introduced selection and information bias. Second, VTE and bleeding risk assessments primarily relied on the VTE system, and their accuracy remained to be verified. Third, limited sample size, follow-up, and sparse events rendered secondary analyses underpowered and statistically fragile, precluding definitive inferences. All findings warrant cautious interpretation, irrespective of statistical significance. Fourth, sequential allocation introduced temporal confounding. Although no concurrent VTE-specific initiatives were identified and non-intervention departments showed no parallel improvement over the same period, secular trends, seasonal case-mix variation, and institutional learning might still confound the intervention effect. PSM balanced individual baselines and mitigated selection bias but could not address group-level temporal bias. And the single-center design constrained the external validity and generalizability of the present findings. Finally, formal quantitative scoring of intervention fidelity was not conducted in this study, which remained a methodological limitation. Future efforts should focus on refining the pharmacist management model throughout the entire VTE prevention and treatment process, with prospective, multicenter studies of larger sample sizes and more rigorous designs needed to validate this model.

## Conclusion

The pharmacist-participated VTE management model was associated with improved implementation rates of mechanical and standardized prophylaxis, generating hypotheses about its potential utility in optimizing quality control for in-hospital VTE prevention and management, which remain to be validated through concurrent controlled trials.

## Data Availability

The original contributions presented in the study are included in the article/Supplementary Material, further inquiries can be directed to the corresponding author.
