# Supplementary material for: The role of pharmacists in quality management of venous thromboembolism: a retrospective, observational, single-center study in the cardiothoracic surgery department
Source: Front Pharmacol. 2026 Jun 26;17:1819628. doi: 10.3389/fphar.2026.1819628 (PMC13352113; doi:10.3389/fphar.2026.1819628)
Supplement: Supplementary file 1 [file Table1.DOCX]

**Supplementary Table 1. Calculation Method for the Outcome Indicator**

| **Outcome Indicator** | **Calculation Method** |
| --- | --- |
| **Primary outcome measure** | |
| Drug prevention implementation rate | Total number of discharged patients with medication orders for prophylaxis / Total number of discharged patients with moderate-to-high VTE risk and low bleeding risk × 100% |
| Mechanical prevention implementation rate | Total number of discharged patients with mechanical prophylaxis orders / Total number of discharged patients with moderate-to-high VTE risk × 100% |
| Combined prevention implementation rate | Total number of discharged patients who received joint prevention orders / The total number of discharged patients with high risk of VTE and low bleeding risk × 100%. |
| Standardized prevention implementation rate | Total number of patients with moderate-to-high VTE risk who received standardized VTE prophylaxis orders / Total number of discharged patients with moderate-to-high VTE risk × 100% |
| **Secondary outcome measures** | |
| Hospital-associated VTE incidence | Total number of discharged patients with hospital-associated VTE / Total number of discharged patients during the same period × 100% |
| major bleeding events incidence | Incidence of bleeding events = Total number of discharged patients experiencing major bleeding during hospitalization / Total number of discharged patients receiving anticoagulant/thrombolytic therapy for prevention of hospital-associated VTE × 100%. |
| Hospital-associated VTE mortality | Total number of patients who died from hospital-associated VTE / Total number of patients discharged during the same period × 100% |
