# Supplementary material for: The role of pharmacists in quality management of venous thromboembolism: a retrospective, observational, single-center study in the cardiothoracic surgery department
Source: Front Pharmacol. 2026 Jun 26;17:1819628. doi: 10.3389/fphar.2026.1819628 (PMC13352113; doi:10.3389/fphar.2026.1819628)
Supplement: Supplementary file 2 [file Table2.DOCX]

**Supplementary Table 2. Balance verification of patients before and after propensity score matching**

| **Comparison of Different Groups** | **Age** | | **Sex** | | **Risk assessment levels of VTE** | | **Risk assessment level of bleeding** | | **Prepare to perform the surgery** | | **Arrhythmia** | | **Hypertension** | | **Heart failure** | |
| --- | --- | --- | --- | --- | --- | --- | --- | --- | --- | --- | --- | --- | --- | --- | --- | --- |
|  | **SMD** | **p Value** | **SMD** | **p Value** | **SMD** | **p Value** | **SMD** | **p Value** | **SMD** | **p Value** | **SMD** | **p Value** | **SMD** | **p Value** | **SMD** | **p Value** |
| **Before PSM** | | | | | | | | | | | | | | | | |
| Baseline group vs. Pilot intervention group | 0.1 | 0.014 | 0.1 | 0.249 | 0.1 | 0.320 | 0.0 | 0.967 | 0.2 | 0.006 | 0.0 | 0.902 | 0.1 | 0.073 | 0.1 | 0.046 |
| Baseline group vs. Pharmacist intervention group | 0.1 | 0.065 | 0.0 | 0.861 | 0.1 | 0.371 | 0.0 | 0.795 | 1.0 | <0.001 | 0.1 | 0.061 | 0.1 | 0.187 | 0.1 | 0.255 |
| Pilot intervention grou vs. Pharmacist intervention group | 0.0 | 0.604 | 0.1 | 0.159 | 0.1 | 0.045 | 0.0 | 0.749 | 1.0 | <0.001 | 0.1 | 0.059 | 0.2 | 0.001 | 0.2 | 0.001 |
| **After PSM** | | | | | | | | | | | | | | | | |
| Baseline group vs. Pilot intervention group | 0.1 | 0.067 | 0.0 | 0.720 | 0.0 | 0.470 | 0.0 | 0.660 | 0.2 | 0.052 | 0.1 | 0.267 | 0.0 | 0.544 | 0.0 | 0.534 |
| Baseline group vs. Pharmacist intervention group | 0.0 | 0.748 | 0.0 | 0.892 | 0.1 | 0.333 | 0.0 | 0.934 | 0.2 | 0.107 | 0.1 | 0.328 | 0.0 | 0.983 | 0.0 | 0.773 |
| Pilot intervention group vs. Pharmacist intervention group | 0.1 | 0.167 | 0.0 | 0.846 | 0.1 | 0.105 | 0.0 | 0.633 | 0.1 | 0.235 | 0.0 | 0.958 | 0.0 | 0.562 | 0.1 | 0.390 |

SMD: standardized mean difference; PSM: propensity score matching.
