# Supplementary material for: The role of pharmacists in quality management of venous thromboembolism: a retrospective, observational, single-center study in the cardiothoracic surgery department
Source: Front Pharmacol. 2026 Jun 26;17:1819628. doi: 10.3389/fphar.2026.1819628 (PMC13352113; doi:10.3389/fphar.2026.1819628)
Supplement: Supplementary file 3 [file Table3.DOCX]

**Supplementary Table 3. Pharmacist involvement in reviewing and implementing VTE prevention measures**

| **Date** | **Count of patients with moderate-to-high VTE risk, n** | **Frequency of pharmacist reviews for patients with moderate-to-high VTE risk, n** | **Count of patients with moderate-to-high VTE risk receiving pharmacist review, n** | **Frequency of pharmacist-initiated communications, n** |
| --- | --- | --- | --- | --- |
| Mar-25 | 222 | 355 | 124 | 69 |
| Apr-25 | 191 | 274 | 126 | 86 |
| May-25 | 204 | 454 | 134 | 127 |
| Jun-25 | 223 | 410 | 163 | 117 |
| Total | 840 | 1493 | 547 | 399 |
