# Supplementary material for: The role of pharmacists in quality management of venous thromboembolism: a retrospective, observational, single-center study in the cardiothoracic surgery department
Source: Front Pharmacol. 2026 Jun 26;17:1819628. doi: 10.3389/fphar.2026.1819628 (PMC13352113; doi:10.3389/fphar.2026.1819628)
Supplement: Supplementary file 4 [file Table4.DOCX]

**Supplementary Table 4. The quality of VTE prophylaxis in concurrent non-intervention departments during the study period**

| **Hepatobiliary surgery** | | | | | | | |
| --- | --- | --- | --- | --- | --- | --- | --- |
| **Indicators** | **Baseline period**  **(n=1125)** | **Pilot intervention period**  **(n=1279)** | **Pharmacist intervention period**  **(n=1063)** | **Chi-square value** | ***P*** value | **Cramér's V, 95% CI** | **Corrected *P*-value** |
| **Pharmacological prophylaxis implementation rate(%)** | 57.3 | 42.5 | 44.0 | 38.340 | <0.001 | 0.132, 95% CI [0.094, 0.174] | <0.001 |
| **Mechanical prophylaxis implementation rate(%)** | 72.3 | 52.8 | 57.3 | 63.536 | <0.001 | 0.170, 95% CI [0.132, 0.213] | <0.001 |
| **Combined prophylaxis implementation rate(%)** | 53.7 | 40.6 | 43.7 | 10.016 | 0.007 | 0.112, 95% CI [0.048, 0.182] | 0.234 |
| **Standardized prophylaxis rate(%)** | 77.3 | 56.7 | 59.7 | 78.344 | <0.001 | 0.189, 95% CI [0.150, 0.227] | <0.001 |
| **Urology** | | | | | | | |
| **Indicators** | **Baseline period**  **(n=575)** | **Pilot intervention period**  **(n=756)** | **Pharmacist intervention period**  **(n=745)** | **Chi-square value** | ***P*** value | **Cramér's V, 95% CI** | **Corrected *P*-value** |
| **Pharmacological prophylaxis implementation rate(%)** | 50.6 | 56.9 | 57.4 | 4.353 | 0.113 |  |  |
| **Mechanical prophylaxis implementation rate(%)** | 86.7 | 87.6 | 88.8 | 0.846 | 0.846 |  |  |
| **Combined prophylaxis implementation rate(%)** | 49.7 | 58.6 | 52.7 | 2.816 | 0.245 |  |  |
| **Standardized prophylaxis rate(%)** | 73.4 | 79.4 | 78.1 | 4.212 | 0.122 |  |  |
| **Neurosurgery** | | | | | | | |
| **Indicators** | **Baseline period**  **(n=251)** | **Pilot intervention period**  **(n=278)** | **Pharmacist intervention period**  **(n=248)** | **Chi-square value** | ***P*** value | **Cramér's V, 95% CI** | **Corrected *P*-value** |
| **Pharmacological prophylaxis implementation rate(%)** | 47.1 | 38.4 | 25.9 | 12.477 | 0.002 | 0.172, 95% CI [0.101, 0.262] | 0.068 |
| **Mechanical prophylaxis implementation rate(%)** | 68.2 | 74.2 | 64.3 | 3.105 | 0.212 |  |  |
| **Combined prophylaxis implementation rate(%)** | 48.8 | 40.7 | 26.3 | 7.100 | 0.029 | 0.180, 95% CI [0.080, 0.311] | >0.999 |
| **Standardized prophylaxis rate(%)** | 68.7 | 62.7 | 61.4 | 2.340 | 0.310 |  |  |
